# Distribution of Prizes and Opening of the New Pathological Institute, London Hospital

**Published:** 1901-07-13

**Authors:** 


### DISTRIBUTION OF PRIZES AND OPENING OF THE NEW PATHOLOGICAL INSTITUTE, LONDON HOSPITAL.

Amid enthusiastic applause from fellow-students and a distinguished assembly of friends, the prizes to successful students and nursing probationers of the London Hospital were presented on Wednesday afternoon by Sir Henry Enfield Roscoe, Vice-Chancellor of the University of London, in the Library of the Medical College. The Letheby Prize in Chemistry was divided equally (£10 each) between Mr. F. W. Daniels and Mr. T. D. Smith; and in presenting these, Sir Henry said it gave him special pleasure, since chemistry was a subject about which he should know something. He recalled the state of things in medical schools of forty or fifty years ago, when chemistry was taught by the Latin master in the intervals of correcting Latin exercises. He knew of a case in which a student, being asked to say to what animal a given skull belonged, and on what it was accustomed to feed, replied that from the appearance of the teeth he should say that the diet was "a mixed one." The skull was that of a sheep. Nothwithstanding the inadequacy of opportunity, however, he looked back upon his student days as some of the happiest of his life. He wished to impress upon the students of the London Hospital the enormous changes that had since taken place; it lay with them to uphold the honour of the profession, which was both an arduous and a noble one, as well as the glory of the London Hospital. Sir Henry spoke a few words of congratulation to each of the prize-winners; the names of the students being announced by Mr. Munro Scott, warden of the college, and those of the probationers by the Hon. Sydney Holland, who alluded to the recent additions to the hospital, and the munificent donations of several benefactors. The number of patients last year would extend, two abreast, from Whitechapel to Rugby; they had used during the year 12 miles of eggs, 9 miles of sticking-plaster, and over 100 miles of lint; 3,000 leeches had been ordered from Spain for the coming year; 30,000 pills would be required, and 10,000 cod-fish to produce the required amount of cod-liver oil. He asked the visitors to spare a little time to go through the wards, and lighten for a few minutes the burden of the poor and suffering.

A vote of thanks was proposed to Sir Henry Roscoe, by Mr. Mansell-Moullin, and, after a brief reply, the gentlemen present adjourned to the new Pathological Institute, which Sir Henry Roscoe declared open, while the ladies went to the garden, where music and refreshments were provided.

